# The catastrophic out-of-pocket health expenditure of multiple sclerosis patients in Iran

**DOI:** 10.1186/s12913-021-06251-4

**Published:** 2021-03-20

**Authors:** Farid Gharibi, Ali Imani, Koustuv Dalal

**Affiliations:** 1 Food Safety Research Center (salt), Semnan University of Medical Sciences, Semnan, Iran; 2 Health Economics Department, Tabriz Health Service Management Research Center, Tabriz University of Medical Sciences, Tabriz, Iran; 3 School of Health Sciences, Mid Sweden University, Sweden and Faculty of Medicine and Healthcare, al-Farabi Kazakh National University, Almaty, Kazakhstan

**Keywords:** Multiple sclerosis, Out-of-pocket payment, Catastrophic health expenditure, Poverty caused by disease

## Abstract

**Background:**

The present study was designed and conducted to evaluate multiple sclerosis (MS) treatment costs and the resulting economic impact imposed on MS patients in Iran.

**Methods:**

This was a cross-sectional study, among randomly selected 300 MS patients, registered in the MS Association of East Azerbaijan Province, Iran (1 year after their treatment began). The regression analysis, ANOVA, T-test, and chi-square were used.

**Results:**

The average amount of out-of-pocket payments (OOPs) by MS patients during the previous year was 1669.20 USD, most of which was spent on medication, rehabilitation care, and physician visits. Their mean annual income was 5182.84 USD. Fifty four percent of families with an MS patient suffer from catastrophic health expenditure (CHE) and 44% experience poverty caused by the OOPs. Occupational status, having supplemental health insurance, and being residents of Tabriz significantly affect OOPs, CHE, and the resulting poverty (*P* < 0.05).

**Conclusion:**

The catastrophic financial burden of health care costs on MS patients and their families justifies health policymakers to promote pre-payment systems and provide subsidies to less well-off patients to protect them from the unfairness of OOPs and its resulting CHE and poverty.

## Background

Multiple sclerosis (MS) is a complicated, inflammatory disease affecting the central nervous system, with its prominent feature, myelin degradation and loss of neuronal axons, resulting in functional problems and disability [1–3]. MS results in several disorders such as cognitive impairment, pain, fatigue, depression, anxiety and various neurological problems [4]. MS is the second most common cause of neurological disability in working-age adults, and 50 to 80% of patients lose their job ten years after disease onset [5]. Unemployment will have severe adverse effects on the patients’ social relations, mental and physical health [6, 7].

Unemployment is the most destructive consequence of MS, the extent of which depends on the duration of the disease, type of the disease, level of neurodegeneration, amount of fatigue, ability to work, level of learning and memory, and the level of stability in the personality traits of patients [8]. Although more than 90% of patients have a job before diagnosis [9], 70–80% of them lose their jobs, 5 years after diagnosis [10]. Statistics show that 30 to 40% of patients’ working days are lost on average due to sickness absence [1]. Considering the high prevalence of illness [11], its early occurrence in the years of productivity, especially the third and fourth decades of life [12], the long-term survival of patients after diagnosis [13] and, ultimately, the destructive effects of the disease on the lives of patients and their families, it is evident that MS is one of the major problems in public health [14].

Literature indicates that 2.3 million people worldwide suffer from MS, increasing every year [15]. The prevalence of MS in Iran was about 15 to 30 cases per 100,000 people in 2011, with direct and indirect costs estimated at $ 24,475 per patient [16]. In Europe, outpatient care costs now constitute 80–90% of the MS-related healthcare costs [17]. The main problem is that many patients avoid receiving essential healthcare because of the high amount of OOPs. Other people who somehow manage the OOPs suffer from poverty and its widespread consequences [18, 19].

Given the high OOPs incurred by MS patients due to the disease’s specific nature and considering the severe economic impact of the MS patients and their families, a study in Iran is warranted. The present study was designed and conducted to evaluate the OOPs of multiple sclerosis (MS) treatment and the resulting economic impact on MS patients in Iran.

## Methods

### Participants

This was a cross-sectional study. The study was conducted during April and May 2018. The data were collected from 300 MS patients registered at a MS patient association (the center which delivered supportive services for all patients with MS in a province) of the East Azerbaijan province and received at least 1 year of MS treatment. The sample size was also determined based on the total number of registered MS patients in the province (1200) using the Morgan table [20]. The randomized sampling method was used for assuring the representativeness of the participants. So, a code was determined for registered MS patients from 1 to 1200 randomly. Then, the first number was determined from 1 to 5 randomly and the next participants were determined by adding three (the sample interval obtained by dividing 1200 by 300). If a participant had not consented to participate, the next number was interviewed.

### Study tool

This study used a questionnaire to collect data on the OOPs by the patients and their families (direct medical cost), other living expenses of the patients and their families, and the demographic and contextual variables. The questionnaire was validated. Ten experts evaluated the content and face validity of the questionnaire. All five aspects of relevance, transparency, simplicity, necessity, and measurability were considered. The average score of the necessity index (Content Validity Ratio or CVR) was assessed and its questions were validated. Then, the mean of the other four indices (Content Validity Index or CVI) was validated. A 70 % acceptance score was considered as the criterion as recommended by the ten experts. In this study, CVR and CVI scores were calculated as 88.5 and 92%, respectively.

### Calculations

Direct medical costs include the costs related to diagnosing, treating, and rehabilitating MS. Direct medical costs constitute the basis for calculating OOPs, and consequent results of poverty [21, 22]. Spending at least 40% of family financial capacity (non-food costs) on OOPs is regarded as CHE [23], and if the share of OOPs exceeds 50% of non-food costs, this will result in poverty caused by healthcare expenditure [24]. Cut-off points were used to judge CHE’s occurrence or non-occurrence and MS disease-related poverty [23–25].

### Statistical tests

In the descriptive analysis of data, the obtained results were reported as mean (standard deviation) and frequency (percent) for quantitative and qualitative variables, respectively. Initially, the OOPs were estimated in Iranian Rials (IRR) and then to USD, using the exchange rates announced by the Central Bank of Iran in US dollars (1 USD = 42,000 IRR). Based on variable type, the regression analysis, ANOVA, t-test, and chi-square were used to investigate the statistical association between demographic and contextual variables with the amount of OOPs, CHE, and the resulting poverty.

For assessing the statistical relationship between OOPs (as a quantitative dependent variable) with demographical and contextual variables the t-test (for qualitative two-state demographic/contextual variables including gender, having supplemental insurance, and being native/resident of Tabriz), ANOVA (for qualitative multi-state demographic/contextual variables including marital status, educational level, occupational status, and type of basic insurance) and regression (for quantitative demographic/contextual variables such as age, age of the patient at disease incidence, duration of disease) were used. Also, for assessing the statistical relationship between CHE and poverty (as qualitative dependent variables) with demographical and contextual variables the chi-square (for all of qualitative demographic/contextual variable including gender, marital status, educational level, occupational status, type of basic insurance, supplemental insurance, and native/ resident of Tabriz) and t-test (for quantitative demographic/contextual including age, age of patient at disease incidence, and duration of disease) were used. All analyses were performed using SPSS19 (*P* < 0.05).

### Ethical considerations

The researchers considered all ethical codes such as patients’ freedom to accept or refuse participation in the study, getting informed consent from all participants, respecting patient privacy, and assuring patients that the results would be used only for the mentioned purposes. Also, the Ethics Committee of Tabriz University of Medical Sciences approved this study (IR.TBZMED.REC.1396.101).

## Results

The mean age of the MSpatients in the current study was 27.18 (±7.64), and 9.95 (± 7.06) years were passed since the disease’s detection. These patients were mostly married, housekeeper, native (residents of) of Tabriz city, and with low education. All patients had basic insurance. Also, most patients were covered by social welfare (Tamine Ejtemaei, 64.3%), but only one-third of the patients had supplementary insurance.

### Out-of-pocket payments (OOPs)

The average OOPs for patients in the last year was 70,106,490 Rials (1669.20 USD) which is spent on doctor visits, diagnostic services, rehabilitation services, medication, hospitalization, home care, complementary and alternative therapies, and unofficial payments to health care providers. Meanwhile, pharmaceutical and informal payments accounted for the highest and lowest share of OOPs, respectively (Table 1).

**Table 1:** The OOPs incurred by patients with MS in the last year

Category	Cost amount (IRR)		Cost amount (USD)	
	Mean	SD	Mean	SD
Physician visit	2,519,500	1,745,300	59.97	41.55
Diagnosis	4,727,000	5,804,350	112.54	138.19
Rehabilitation	9,481,830	99,625,340	225.75	2372.03
Medicine	50,757,550	61,977,004	1208.51	1475.64
Hospitalization	2,010,200	4,062,930	47.86	96.73
Home care (medical)	328,660	4,866,630	7.82	115.87
Complementary/alternative therapies	163,330	1,267,360	3.88	30.17
Informal paid	118,400	629,500	2.81	14.98
Total	70,106,490	121,767,380	1669.20	2899.22

### Income

The patients’ average annual income and their family was 217,679,590 (±192,808,420) Rials, an equivalent of 5182.84 (±4590.67) USD. Their median income was 180 million Rials (4285.71 USD). On average, MS patients’ families spent 68% of their annual income from non-food costs and 32% from food costs.

### Negative consequences of the unfairness of OOPs

The results indicate that 54% of families with MS patients suffer from CHE and 44% experience poverty resulting from the MS treatment OOPs. The majority of the MS patients acknowledged that, due to the high healthcare expenses, in many cases (73%), they delayed the treatment or had to use low-quality care (84%). More than half of the MS patients delayed or refused to access essential care due to distance problems as they live far away from their nearest MS treatment centres. About 17% of them relocated their house to have easier access to the needed MS care. The majority of patients had been forced to obtain grants or loans from others because of the high OOPsof the illness, often from relatives and acquaintances (not from the government and non-governmental support centres). The majority of the MS patients (58%) also consider the disease’s financial burden is very high and out of their financial ability (Table 2).

**Table 2:** Negative effects of costs on MS patients and their families

Variable		Frequency	Percent
Catastrophic health expenditure	Yes	162	54
	No	138	46
Poverty resulted from the costs	Yes	132	44
	No	168	56
Postpone the treatment or refuse it due to high costs	Yes	219	73
	No	81	27
Using low quality care since quality care is expensive	Yes	254	84.3
	No	47	15.7
Postpone or refuse to use health care due to far distance between the patient’s living place and care providing centers	Yes	171	57
	No	129	43
Patients had to move their living place to have access to care center	Yes	52	17.3
	No	248	82.7
Patients had to borrow money from their relatives to pay for their care	Yes	228	76
	No	72	24
The source of the money used to pay for care costs	First-degree relatives	109	49.8
	Third-degree relatives and acquaintances	97	44.3
	Supportive organizations and charity	13	5.9
The financial burden on the patient and his/her family due to high costs of care	Very low	24	8
	Somewhat	45	15
	High	57	19
	Very high	174	58

### Multivariable analyses

Occupational status, having supplemental insurance, and being a resident of the city had a significant effect on OOPs, CHE and poverty. CHE are very low for public-sector employees, the average for pensioners and homemakers, and very high for the unemployed and students. MS patients ranges with supplemental insurance had significantly lower OOPs and less poverty than those without supplemental insurance. Resident patients of Tabriz city also had significantly lower OOPs (*P* < 0.05) (Tables 3 and 4).

**Table 3:** Significance of the relationship between demographic and contextual variables with MS healthcare costs and their effects

Independent variables (demographic and contextual)	Dependent variables (costs imposed and their effects)	Significance of relationship(***P***-value)
Age	The amount of OOPs	0.328
	Catastrophic health expenditure	0.217
	Poverty resulted from costs	0.322
Gender	The amount of OOPs	0.836
	Catastrophic health expenditure	0.123
	Poverty resulted from costs	0.376
Marital status	The amount of OOPs	0.204
	Catastrophic health expenditure	0.176
	Poverty resulted from costs	0.065
Educational level	The amount of OOPs	0.951
	Catastrophic health expenditure	0.248
	Poverty resulted from costs	0.528
Occupational status	The amount of OOPs	0.706
	Catastrophic health expenditure	0.007
	Poverty resulted from costs	0.059
Type of basic insurance	The amount of OOPs	0.591
	Catastrophic health expenditure	0.403
	Poverty resulted from costs	0.243
Supplemental insurance	The amount of OOPs	0.825
	Catastrophic health expenditure	0.007
	Poverty resulted from costs	0.030
Resident(in Tabriz)	The amount of OOPs	0.038
	Catastrophic health expenditure	0.417
	Poverty resulted from costs	0.465
Age of patient at disease incidence	The amount of OOPs	0.396
	Catastrophic health expenditure	0.835
	Poverty resulted from costs	0.984
Duration of disease (years)	The amount of OOPs	0.389
	Catastrophic health expenditure	0.594
	Poverty resulted from costs	0.667

**Table 4:** Significance of the relationship between quantitative demographic and contextual variables with OOPs (regression results)

Independent variables (demographic and contextual)	Unstandardized Coefficients		Standardized Coefficients	Sig.
	B	Std. Error	Beta	
Age	71,257.014	72,746.883	0.057	.328
Age of patient at disease incidence	90,342.635	88,561.430	0.522	.396
Duration of disease (years)	85,943.286	99,720.575	0.050	.389

## Discussion

This study aimed to investigate the CHE and poverty related to OOPs among MS patients in Iran. The study results show that 54% of families with MS patients incur CHE and 44% of families experience poverty resulted from the healthcare OOPs of the MS. MS patients survive more than other chronic diseases, while MS patients have significantly reduce their workability and productivity due to gradually occurring disabilities [26, 27]. MS is a chronic and disabling disease and demonstrating the same trend in Iran, such as cancer [25].

Other studies in Iran show that CHE’s amount paid by families with MS patients from 3.37% in Ahvaz [28] to 20.6% in Sanandaj [15]. So, the rate of CHE in East Azerbaijan is higher than in Ahvaz and Sanandaj. Differences could be due to education level, job status, patient insurance status, social welfare level, and even community culture [29]. Part of the observed difference may also be due to differences in computational methods or comprehensiveness of study tools used to assess all parts of patients and their families’ incurred cost. For example, based on these two items, the amount of health care CHE in Brazilian households varies from 0.7 to 21% [28] and in Kenya from 1.52 to 28.38% [30]. Although the approach and thresholds were the same in the current study and other studies done in Iran (WHO approach and threshold of 40%), the assessing of other studies showed that instruments were not comprehensive in terms of cost categories and cost items in each category.

The current study has also shown that the financial burden caused by the MS due to the high amount of OOPs is such that most patients do not receive essential care or prefer delayed care, receive poor quality care, and request loans to cover the OOPs. More than half of the MS patients had a history of delayed or even inadequate access to essential care due to the long distance between their home and care centre. It is an interesting note here that 17% of MS patients and families had to relocate due to the distance problem. Also, 77% of the MS patients found that the MS disease’s financial burden is very high and unbearable. A similar study showed that 11.8% of MS patients lack access to the usual sources of public health services; 31% fail to see a specialist; 10.5% have severe problems with purchasing the needed medicines; 4.1% face significant problems related to accessing their needed care; 2.4% are unable to receive mental health care [31]. The financial burden of MS and its induced problems was higher in Iran. This is related to the lower social protection against MS costs in Iran because of the poor basic health insurance and their weak coverage of disease costs. It is also related to the high price of existence supplementary insurance in Iran, so that most patients with MS and their families cannot pay their premium and the government and health system did not have any subsidies in this regard.

The critical point to be mentioned in this study is that although all patients had basic health insurance, they could still not cover CHE and cope with the resulting poverty. In the United States, although most patients with MS have health insurance, only half the cost of care is covered by insurance [32]. Also, in China, the CHE are so high that health insurance cannot cover it significantly [33]. In Iran, OOP is the primary way of financing the health system. So the amount of health care OOPs and associated CHE and poverty is relatively high. Accordingly, reducing OOPs to less than 30% and CHE to 1% is one of the goals of Iran’s health care system [28]. The literature showed that 0.88% of Iranian population faced poverty due to CHE [34]. Therefore, it is necessary to reduce the amount of OOPs, especially among patients with chronic and disabling diseases such as MS, when receiving health care through the use of appropriate insurance systems [35]. For example, in Turkey, direct OOPs dropped from 27.6% in 2000 to 19.3% in 2006, with a declining trend. As a result, the amount of health care OOPs in Turkey is only 0.6% due to adequate insurance system [35].

This study showed that occupational status, supplemental insurance, and being residents of Tabriz city have significant association with the amount of OOPs, CHE, or the resulting poverty. Variables such as the brand of medicines used by patients, housekeeping, income, and having health insurance were found to have a statistically significant relationship with CHE of MS [28]. Also, variables such as patients’ financial status, level of education, having supplementary insurance, other diseases in the individual and their families, living in rural areas, and using other health services such as dentistry and rehabilitation have a relationship with CHE of MS [15]. The literature suggests that protecting the patients’ community against the CHE is one of the underlying concepts of many of the health system’s functional indicators such as justice, equality and access to health care [36]. However, the Iranian health system has not been successful in protecting citizens and MS patients in particular.

Another point to note here is that diseases, especially chronic and debilitating diseases such as MS, can impose extra costs on patients and their families, including direct non-medical costs (appropriating the workplace and home environment, costs of visiting care centres), as well as indirect costs (absenteeism or unemployment). Calculating these costs, along with OOP expenses, can quantify the financial burden of MS.

The policy makers’ practical points of action could be widening the scope and depth of basic health insurance coverage for essential health care, granting financial support and proper subsidies for expanding supplementary health insurance. MS patients and their families could be assessed and ranked according to their CHE and healthcare cost-directed poverty. In the next stage, using that ranking, MS patients and their families can receive supportive services from governmental and charity organizations, procurement of healthcare services by the health system, especially medicine cost. Assuring a close relationship between the ministry of health and MS association should be ensured. Considering the current economic situation in Iran, charity organizations, international organizations such as World Health Organizations (WHO) and World Bank should be involved in financial and better procurement of MS medicine, appropriate, timely diagnosis and rehabilitation technologies and devices. Assessment of the MS patients’ ability level and employing them accordingly may provide necessary economic support to the MS patients and their families.

One of the limitations of the present study is that the researchers could not meet some of the MS patients because they did not accept to participate in the study due to their poor health condition and poor mobility. It is important to note here that their participation in the study would likely increase the estimated OOPs, the CHE and the resulting poverty. They were unable to earn and pay for health care costs and imposed more costs on their families’ budget. This problem could be solved in future studies by creating a comprehensive electronic record system of the MS patients, where all the care and rehabilitation information and costs are recorded. Perhaps creating such an electronic system could eliminate the need to collect data directly from the patients to estimate CHE’s occurrence and get an overall scenario of the MS victims.

Another limitation, which may be emerging in all CHE studies, is related to the questionnaire’s comprehensiveness and accuracy used to collect data [37]. If the questionnaire is not calculated and considers all the costs imposed on patients and their families, the final estimate can be considered less than the actual amount and the results of the study have low validity. This problem was solved in the current study by developing a comprehensive questionnaire approved by ten experts. Simultaneously, the data collectors’ proper training on how to ask questions and how to enter patient answers into the relevant questionnaire was assured in the present study.

Another limitation, which is generally present in CHE studies is recall bias [37]. If the patient cannot remember all the incurred costs on himself and his/her family, the CHE can be underestimated. Developing and using a comprehensive electronic health record can also solve this problem. However, if the electronic health record is not used in a health system, many of the incurred costs on patients and families can still be obtained by referring to the documents and clinical records. This was the approach taken in our study. Many of the costs imposed on patients can be extracted by referring to the medical and financial documents and records of the healthcare providers to ensure the correctness of the patient’s cost amount. The crucial points are that in chronic diseases such as MS, the delivered healthcare and other costs are often repetitive. The healthcare provisions are often received from specific healthcare centers, which may hinder timely access to services and transport costs.

## Conclusion

The present study showed that the high amount of OOPs among MS patients in Iran has led to severe and unbearable financial pressure. The health system’s existing financial support and insurance have failed to meet their expected financial protection. The Iranian health care system may promote the quality and fair coverage of health care on MS patients using approaches such as enhancing the level and depth of coverage required by basic and supplemental insurance, using prepaid insurance methods instead of OOPs, establishing comprehensive specialized centres for the care of MS patients, and providing MS patients and their families with material and spiritual support. Findings of the study may also help other low- and middle-income countries in a similar context. The current study can help health policymakers by demonstrating the dimensions of existing problems, providing practical suggestions, and subsequently improving health and satisfying MS patients and their families.

## Data Availability

The study data are available and will send to make accessible by Dr. FaridGharibi (Email: gharibihsa@gmail.com).

## Abbreviations

MS: Multiple Sclerosis
OOPs: Out-of-Pocket Payments
CHE: Catastrophic Health Expenditure
LMICs: Low- and Middle-Income Countries
OECD: The Organization for Economic Co-operation and Development
WHO: The World Health Organization
CVR: Content Validity Ratio
CVI: Content Validity Index
